# Microfluidic Point-of-Care (POC) Devices in Early Diagnosis: A Review of Opportunities and Challenges

**DOI:** 10.3390/s22041620

**Published:** 2022-02-18

**Authors:** Shih-Mo Yang, Shuangsong Lv, Wenjun Zhang, Yubao Cui

**Affiliations:** 1 School of Mechatronic Engineering and Automation, Shanghai University, Shanghai 200444, China; smyang@shu.edu.cn (S.-M.Y.); 1500357613@shu.edu.cn (S.L.); 2 Division of Biomedical Engineering, University of Saskatchewan, Saskatoon, SK S7N 5A9, Canada; wjz485@mail.usask.ca; 3 Clinical Research Center, The Affiliated Wuxi People’s Hospital, Nanjing Medical University, 299 Qingyang Road, Wuxi 214023, China

**Keywords:** microfluidic, POC (point-of-care), medical testing, COVID-19, wearable devices, telemedicine

## Abstract

The early diagnosis of infectious diseases is critical because it can greatly increase recovery rates and prevent the spread of diseases such as COVID-19; however, in many areas with insufficient medical facilities, the timely detection of diseases is challenging. Conventional medical testing methods require specialized laboratory equipment and well-trained operators, limiting the applicability of these tests. Microfluidic point-of-care (POC) equipment can rapidly detect diseases at low cost. This technology could be used to detect diseases in underdeveloped areas to reduce the effects of disease and improve quality of life in these areas. This review details microfluidic POC equipment and its applications. First, the concept of microfluidic POC devices is discussed. We then describe applications of microfluidic POC devices for infectious diseases, cardiovascular diseases, tumors (cancer), and chronic diseases, and discuss the future incorporation of microfluidic POC devices into applications such as wearable devices and telemedicine. Finally, the review concludes by analyzing the present state of the microfluidic field, and suggestions are made. This review is intended to call attention to the status of disease treatment in underdeveloped areas and to encourage the researchers of microfluidics to develop standards for these devices.

## 1. Introduction

The early detection of infection is critical for the management of infectious diseases. The COVID-19 pandemic has revealed that countries must identify and isolate an infection source early to control a disease spread successfully. Moreover, early detection and timely treatment can improve the cure rates and reduce treatment costs for cancer and other serious diseases. Increasingly networked global transportation and rapid changes in the natural environment have increased the impact of illness on public health. Generally speaking, virology, serology, and imaging diagnosis are the most common medical detection methods [1].

Serology identifies deviations in relevant protein markers in the human body, virology detects the presence of viruses in the body, and imaging diagnosis observes whether organs in the human body have morphological lesions. Expensive professional testing instruments must be placed in research laboratories, and their complex operation requires trained professionals. This makes the detection techniques time-consuming, costly, and labor-intensive, limiting their applicability for early disease diagnosis [2].

For people in underdeveloped areas, the lack of medical resources is even more severe. Patients in these areas often do not have enough time or money to receive routine medical testing. For example, in developing countries in Africa or Southeast Asia, basic medical facilities are insufficient. Countries with imperfect health systems and few medical resources are more likely to struggle with infectious diseases [3]. If a highly contagious disease is prevalent in these areas, the source of infection may not be investigated and isolated in time, leading to the epidemic spread of the disease. The COVID-19 pandemic has revealed that an infectious disease outbreak in a single region also affects other countries because of substantial international travel [4].

The World Health Organization (WHO) reported that, for the April 2021 COVID-19 outbreak in India, 26,528,846 people were infected, and nearly 300,000 had died as of 23 May [5]. Although the epidemic in India is already severe, rural areas still have insufficient detection capabilities for COVID-19. COVID-19 may be confused with the common cold because of a lack of nucleic acid detection equipment and little local knowledge of COVID-19. WHO experts thus believe that the number of COVID-19 cases in India has been substantially underestimated. In addition to India, Nepal, Laos, Cambodia, Thailand, and Bhutan bordering India are also facing a new wave of infections. The situation in Southeast Asia is urgent. As of 16 May 2021, the number of confirmed cases in Southeast Asia had reached 2,529,924, and the number of new cases in the previous week accounted for 53% of the world’s new cases [5]. If the situation in India continues to worsen, it may have a significant spillover effect on neighboring countries. Because medical resources are scarce in many Southeast Asian countries, persistent epidemics are difficult to prevent which would affect the society and stability of these countries, while increasing international tension in the region. Compared with the less developed areas, the European region belonging to developed regions is also the most seriously affected area by COVID-19. As of 28 January 2021, there were 32,218,360 cases and 701,991 deaths, making it the continent with the highest mortality rate in the world [6]. Although the developed countries in Europe have excellent medical systems, the rapid spread of COVID-19 has also greatly affected the European region. Therefore, until December 2021, the European region has been strengthening the vaccination of the COVID-19 vaccine to alleviate the impact of the virus on residents [7]. The lack of technologies for the early detection and prevention of major infectious diseases in a country will eventually lead to burdens on the medical systems of the country.

Access to clinical diagnosis is limited in low-resource areas. Even in developed countries, rural areas may have insufficient health screening and disease prevention services [8]. Therefore, developing portable, inexpensive medical inspection equipment for field testing or self-testing at home is of great practical significance. Such devices could alleviate the strain on the medical system in underdeveloped areas, reduce the overuse of medical resources, and allow for early diagnosis. Microfluidic technologies have been developed over the past ten years to solve these problems. The highly integrated microfluidic chips are small, easy to carry, and require few reagents to perform detection. More importantly, microfluidic chips need a shorter detection time than conventional medical detection devices; thus, microfluidic chips are particularly suitable for rapid detection in areas with scarce medical resources. Microfluidic technologies have been used in fields including medical diagnosis [9], laboratory testing [10,11], cell analysis [12], and other biochemical research because of their convenient operation, small size, and portability. Microfluidic technology has also enabled the development of point-of-care (POC) technologies. Substantial progress has been made in applying microfluidic technology in the POC field. A POC test (POCT) is a simple analytical test that can provide patients with a medical diagnosis in real-time, even in environments with limited resources [13]. The application of POCTs has brought hope to developing countries with limited infrastructure and economic development but that still require timely medical detection services [14].

This review focuses on the need for and development of technologies for early detection of diseases in underdeveloped regions. We first describe microfluidic devices and introduce their advantages and types. Then, we introduce the current and future applications of microfluidic POC devices for medical detection of infectious diseases, cardiovascular diseases (CVDs), tumors, and other chronic diseases. Finally, the current commercialization status of microfluidic devices is described and our opinions on the technology are given. Readers of this review will obtain a more comprehensive understanding of the current state of microfluidic technologies for early disease detection. The review may also bring attention to the living conditions of people in underdeveloped areas.

## 2. Microfluidic POC Devices

### 2.1. Introduction

A microfluidic system is a small portable system that can complete sample pretreatment, separation, dilution, mixing, chemical reaction, detection, and product extraction. These systems can increase analysis speed and efficiency as well as reduce the consumption of samples or reagents. Moreover, the process of analysis can be completely automated, eliminating human interference, preventing pollution, and allowing for efficient repeating of experiments. The microfluidic chips used in laboratory medicine are representative of this technology. Microfluidic devices have been widely used in chemistry, biology, physics, engineering, and biomedical sciences. Microfluidic devices are considered mobile devices because a sample analysis can be performed entirely within the small, portable device [15]. The reduced reagent use of microfluidics is also beneficial in areas with scarce resources or challenging terrain. Miniaturization and a low reagent consumption reduce the cost of each experiment, facilitating detection activities in underdeveloped areas [16].

A POCT is a portable device used for analysis and detection outside a conventional laboratory [17]. POCTs can provide early and rapid diagnoses for patients with the disease. The advantages of POCTs are their portability and detection speed. POCT technology can also be used for animal disease detection. Pascual-Garrigos et al., improved the loop mediated isothermal amplification (LAMP) assay to realize the early POC detection of bovine respiratory diseases (BRD), so as to reduce the risk of morbidity and mortality of dairy cows on the farm and reduce the economic burden of farmers. It can be seen that POCT technologies can be widely used in different occasions [18].

Microfluidic systems have had a substantial influence on the applications of POCTs in medical diagnosis. Microfluidic systems have a high sensitivity and can obtain results rapidly; thus, microfluidic systems combined with POCTs are the most portable and least expensive devices for rapid detection [19].

### 2.2. Advantages of Microfluidic POC Devices in Medical Laboratory

The detection of biomarkers is key in medical detection. The conventional detection method for protein biomarkers is an enzyme-linked immunosorbent assay (ELISA). ELISA is an ensemble test that requires enough target particles to generate measurable signals as readings. ELISA is primarily a plate-based assay. The method uses a variety of enzyme substances, such as alkaline enzyme, horse radish oxidase and β-galactosidase. Specific substrates, such as ortho-phenyldiamine dihydrochloride, are used to hydrolyze through the top of the enzyme to produce colored results [20]. ELISA has a high sensitivity in detecting protein content, therefore it is the gold standard method for identifying or quantifying proteins [21].

ELISA can also detect and analyze infectious pathogens such as viruses [22]. ELISA has been successfully used to detect the dengue virus [23], Zika virus [24], influenza virus [25], and numerous other viruses, including the SARS-CoV-2 virus [26,27].

**Figure 1:**
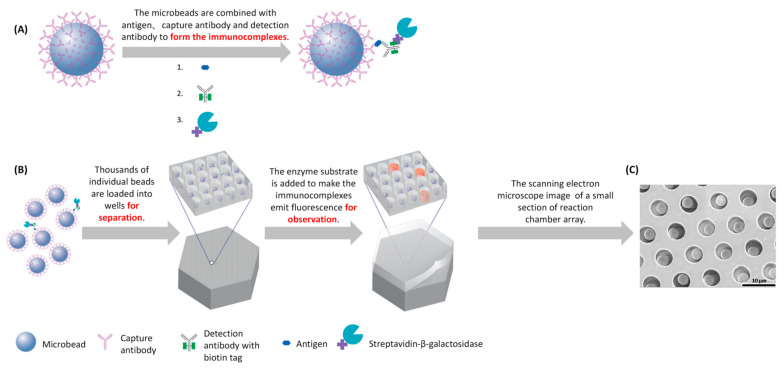
Single-molecule enzyme-linked immunosorbent assays (digital ELISA) based on singulation of enzyme labels. (**A**) Capturing and labeling individual protein molecules on microbeads using standard ELISA reagents. (**B**) Microspheres are loaded into a reaction chamber array for the separation and detection of individual molecules. (**C**) Scanning electron microscope image of the microspheres after placement in the reaction chamber array. Reprinted with permission from [29]. Copyright 2010 Springer Nature.

Conventional ELISA must be improved for it to obtain the high detection sensitivity necessary for protein detection [28]. Rissin et al., reported a method capable of detecting ultra-low protein concentrations. Each protein molecule was captured by microbeads to form a sandwich complex structure, which was deposited in the reaction chamber array. Enzyme labeling was used to detect which microbeads captured a single protein and which beads did not. Single-molecule ELISA (digital ELISA) based on the singulation of enzyme labels can detect proteins with concentrations $<10^{-15}M$ in serum. Digital ELISA uses the same reagents developed for standard ELISA, reducing the development costs. Figure 1 shows the process of digital ELISA [29].

**Figure 2:**
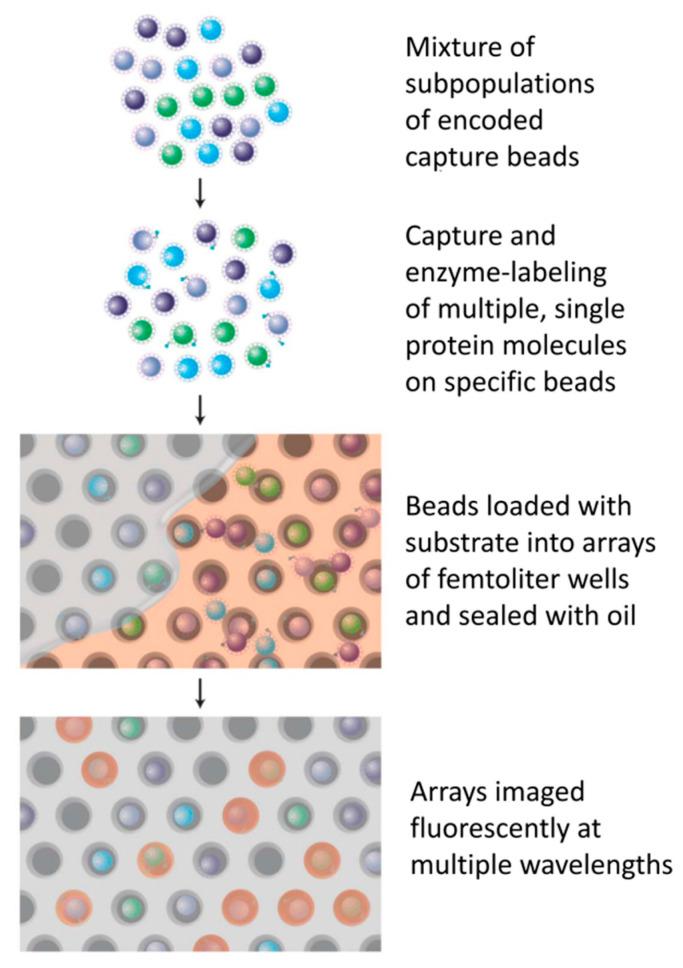
Schematic of multiplexed digital ELISA process. Reprinted with permission from [33]. Copyright 2013 Royal Society of Chemistry.

On the basis of digital ELISA, Chang et al., proposed a theoretical model that generates signals as a function of time, concentration, and binding constant [30]. Kan et al., improved the efficiency of the bead reading by improving the efficiency of the bead hole falling and developed a digital ELISA method that can detect IL-17A with 100% efficiency [31]. In addition, later studies have revealed that capillary force and magnetic force or ionic concentration-polarization (CP)-based biomolecule preconcentration and dielectrophoresis (DEP) can improve the efficiency of bead pore falling, improving the sensitivity of digital ELISA [31,32]. For single-molecule protein detection, Rissan et al., developed multiple single-molecule immunoassays that can simultaneously detect multiple protein molecules that were called multiplexed digital ELISA. In this method, the microbeads are divided into different types, and each type of microbead has different specific antibodies that combine with different protein molecules. Each type of microbead also has different fluorescence characteristics, presenting different fluorescence images to distinguish the different protein molecules, which is shown in Figure 2 [33]. The most common method for multiple molecular detections is flow cytometry [34]. xMAP-based technologies are also a recent research pathway for multiple molecular detection technologies. xMAP-based technologies can simultaneously detect, for example, viruses, bacteria, and proteins and are widely used in pharmaceutical, clinical, and research laboratories [35].

Although digital ELISA can detect multiple protein markers simultaneously and can achieve a higher sensitivity compared with conventional ELISA, these detection methods often require nonportable instruments and are unsuitable for rapid detection, especially in underdeveloped regions. These detection methods must become more widespread, and a method to achieve this goal is microfluidic technologies. Current microfluidic technologies can be combined with other medical detection technologies to achieve better detection results.

Mass spectrometry (MS) based proteomics has become an indispensable technology to interpret the coding information in the genome. Mass spectrometry can identify and accurately quantify thousands of proteins from complex samples [36]. A combination of MS and a microfluidic chip (μchip–MS) can improve the tools currently used by clinicians. It can provide new methods for early detection, diagnosis, monitoring, and the treatment of noncommunicable diseases (such as heart disease, stroke, cancer, and diabetes) [37,38,39]. The emergence of this platform constitutes a new application of microfluidic chips and compared with conventional immunoassay technologies, μchip–MS has a higher sensitivity and specificity in proteomic analysis and requires less labor and time. Surface plasmon resonance (SPR) is a useful method, which can provide optical and label free detection of target analytes. The SPR detection method has many advantages, such as a low production cost, high detection accuracy and sensitivity, and has been successfully applied to cancer biomarkers and virus detection [40,41]. In 2019, Liu et al., used nanoparticle and microfluidic technologies to realize the SPR detection of the target protein. The experimental results were better than those of the single SPR detection method [42], thus, the inclusion of microfluidic technologies in existing technologies can increase the sensitivity of conventional tests.

Numerous conventional medical detection technologies have incorporated microfluidic POC devices. These devices can detect biomarkers in blood and can obtain excellent detection results [43,44]; these characteristics indicate the suitability of microfluidics for disease detection in underdeveloped areas.

### 2.3. Classification of Microfluidic POC Equipment Types

The first microfluidic device was a micro gas analysis system based on gas chromatography developed by Stanford University in the 1970s. The main components were made from silicon and glass by using photolithography and chemical etching technology [45]. Microfluidic chips based on polydimethylsiloxane (PDMS) materials have matured, and current commercial microfluidic devices are made of a PDMS polymer by using soft lithography [46].

Recently, cheap paper and three-dimensional (3D) printing technology have been used for microfluidic chip fabrication, reducing the cost of manufacturing [47,48]. These devices can be used for POC testing in underdeveloped areas. Mobile sensors based on integrated microfluidic devices and smartphones are a successful example of combining microfluidics with POC technologies [49]. Handheld centrifugal microfluidic devices and microfluidic POC devices using DEP technology also constitute special research topics, extending the research into microfluidic POC devices [50].

Microfluidic equipment based on PDMS, paper, and 3D printing and systems integrating microfluidics and smartphones is described in the following sections. Subsequently, and finally, handheld centrifugal microfluidic devices and microfluidic POC deceives using DEP technology are introduced.

#### 2.3.1. Microfluidic Equipment Made of PDMS

PDMS and thermoplastic molding methods are typically used in microfluidic manufacturing [51,52]. The most common commercial microfluidic devices are made of a PDMS polymer. The biggest advantage of the PDMS polymer is its low price, which enables the large-scale production of microfluidic systems. Soft lithography is a non-lithographic method used to copy patterns, simplifying the carving process. It is particularly suitable for fabricating channels in bulk polymers [53]. PDMS and soft lithography technology are critical for the inexpensive production of microfluidic devices [54]. Microfluidics require little infrastructure, are inexpensive, and are easy to manufacture, thus, people can learn to use them at low cost, promoting the continued development of PDMS microfluidic devices.

We needed only to make the template of a PDMS microfluidic chip in a dust-free room to carry out continuous replication and production, which really makes it easier for us to design and manufacture the chip in the early stage of the experimental project; however, PDMS-based microfluidic chips still have some shortcomings. First, utilizing PDMS to produce chips is not suitable due to a great demand for the output of chips in large-scale production. In comparison, the method of making microchannels with thermoplastic elastomers is more convenient and can be applied to large-scale microfluidic chip production. Unlike PDMS, thermoplastic elastomers can be processed using many industrial polymer manufacturing technologies, making them cheaper and easier to be mass-produced. Therefore, in the actual application, the manufacturing of microfluidic chips with thermoplastic elastomers is more widely used [55]. Moreover, PDMS microfluidic chips have a two-dimensional structure. Compared with a three-dimensional structure, the two-dimensional structure is unable to simulate the flow of fluid in the stereoscopic space. Thus, PDMS microfluidic chips require continual improvement [56].

#### 2.3.2. Paper-Based Microfluidic Devices

Paper-based microfluidic systems have become popular in practice due to their low cost, ease of manufacture, and portability. The first paper-based microfluidic device was designed by Whitesides et al., in 2007 [57]. Paper-based microfluidic devices are widely used in POC applications such as public health and environmental monitoring [58].

Because paper is cheap, it is particularly popular for applications requiring a low cost, easy operation, and rapid analysis, such as for disease diagnosis in low and medium resource areas [59]. In addition, patterning technology is the key factor that renders paper-based microfluidic devices usable in the field of POC. The drawing of microchannels on paper-based microfluidics by wax printing can yield control over liquid flow that is comparable with that of standard microfluidics; this has resulted in paper-based microfluidic technology emerging as a new field of microfluidic device-related research [60].

**Figure 3:**
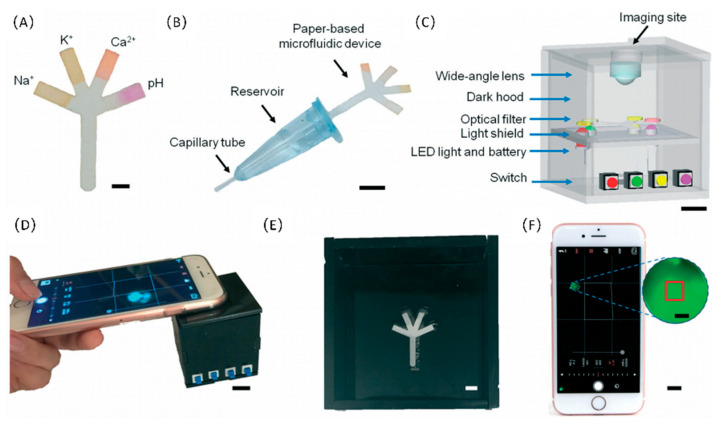
Measurement of electrolytes in tears by a paper-based integrated microfluidic system and data acquisition with a smartphone. (**A**) Paper-based microfluidic device impregnated with fluorescent probe. (**B**) Samples were collected and diluted using capillary tubes. (**C**) The schematic diagram of the portable readout device. (**D**) The use of the portable readout device in combination with a smartphone to capture the image of the fluorescent probes. (**E**) Photograph of the interlayer groove used to place the paper-based microfluidic device. (**F**) Screenshots of smartphone applications that capture the measured images. Reprinted with permission from [62]. Copyright 2017 Royal Society of Chemistry.

Paper-based microfluidic chips can be applied to analyze various analytes in the human body, including urine, saliva, blood, tears, and other bodily or exocrine fluids. These microfluidic chips can also be used for POC detection of diseases [61], for example, the analysis of tear fluid components can be used for the early diagnosis of dry eye disease. Yetisen et al., designed a portable microfluidic control system that can quantitatively analyze the electrolytes in tear fluid. The control system consisted of a paper-based microfluidic device, a portable readout device and a smartphone for data acquisition. In the monitoring process, the operator only needs to put the tear sample in the paper-based microfluidic device and wait for the capillary in the fluorescent probes to absorb the sample. Finally, after putting the fluorescent probes into the portable readout device, the measurement results can be observed with the smartphone. Experiments on samples with different ion concentrations revealed that the system could be used for early POC diagnosis of dry eye disease, which is shown in Figure 3 [62]. For the early detection of acute myocardial infarction (AMI) after the onset of chest pain symptoms, Lim et al., developed a highly sensitive microfluidic paper-based device. The device could simultaneously detect multiple cardiac biomarkers and was effective for both the early and late diagnosis of AMI [43].

The low cost of a paper-based microfluidic device increases its applicability for POC detection in underdeveloped areas; however, if only paper is used as the main detection material, the microfluidic system often cannot compare with the conventional laboratory detection methods for disease detection.

The paper-based microfluidic device may not be able to detect complex samples accurately, but because paper-based microfluidic devices often use a colorimetric method as the basis for detection, it is convenient to be integrated with smartphones as the detection result can be obtained by analyzing the color of the picture. There is no doubt that due to the very low cost of paper, paper-based microfluidic devices would be the key to many fast and real-time detection applications for a period of time to come.

#### 2.3.3. 3D-Printed Microfluidic Devices

In general, 3D printing technologies have influenced the development of microfluidics [63]; these technologies are automated, eliminating the human resources required for manufacturing conventional PDMS microfluidics [64], and have not only reduced costs but also achieved a good resolution and throughput, increasing their recognition by the scientific community [65,66]. High latitude means that the real flow of fluid can be simulated more accurately; thus, microfluidic devices with more dimensions are currently being developed [67]. Furthermore, 3D printing methods have substantial commercial potential and can rapidly produce prototype products, increasing the frequency and efficiency of experiments, and enabling the rapid commercialization of experimental technologies [68,69].

In fact, microfluidic POCT devices based on 3D printing technology have been applied in practical applications. Song et al., developed a structured platform with a built-in microfluidic detection box. The platform uses reverse-transcription loop-mediated isothermal amplification (RT-LAMP) technology to realize the rapid detection of the Zika virus. The detection box is manufactured by 3D printing technology and it does not need to be heated by an external power supply, but through chemical heating to meet the conditions required for the experiment. Only saliva samples were needed to detect the Zika virus [70]. Additionally, Kadimisetty et al., developed a microfluidic POCT device with a simple operation and low cost for nucleic acid amplification tests to diagnose infectious diseases [71].

Three-dimensional printing technology has an unprecedented processing capability, providing new opportunities in microfluidic POCT by accelerating experiments and the commercialization of the results. Although 3D microfluidic chips are still less precise relative to traditional PDMS microfluidic chips, 3D printing technology is useful for producing microfluidic POCTs. Due to the rapid manufacturing capacity of 3D printing technology, large-scale 3D-printed microfluidic equipment production has become possible. In this approach, 3D-printed microfluidic POC devices can play a significant role in underdeveloped areas.

#### 2.3.4. Mobile Sensors Based on Integrated Microfluidic Devices and Smart Phones

The continual development of microelectronics since the beginning of the 21st century has resulted in smartphones; these handheld devices now have a processing power equivalent to that of a small computer and can smoothly complete simple data processing tasks. Smartphones can replace conventional computers for data processing, computing, and data collection in underdeveloped areas [72,73,74]. In particular, combining smartphones with microfluidic devices is a comprehensive solution for the new generation of mobile sensing applications, known as MS^2^. Portable mobile sensors based on microfluidic devices integrated with smartphones are useful for disease detection in remote areas.

The Zika virus is transmitted by infected mosquitoes and can cause fever, headache, rash, and joint muscle pain, especially for newborns, making timely detection necessary. Kaarj et al., developed a wax paper microfluidic chip using reverse transcription loop-mediated isothermal amplification (RT-LAMP). The color change induced by ZIKV RNA in the microfluidic detection area is observable within 15 min, and virus detection can be completed with a smartphone camera [75]. Urine also contains many markers that can be used to detect disease. Jalal et al., developed a microfluidic device combining reagent paper and polycarbonate (PC) plastic materials. A smartphone camera can capture colorimetric changes after the reaction of the analyte, and algorithms were designed to detect the chemical components in urine. Thus, diagnosis can be achieved based on urine composition [76].

MS^2^ applications are easy to use, enabling untrained users to obtain reliable test results and we believe that MS^2^ is a valuable and promising technology; however, many difficulties remain in this emerging field. More complex detection experiments require the introduction of experimental samples into existing MS^2^ systems as well as additional interface equipment for the detection. Therefore, applications of MS^2^ still require improvement in MS^2^ devices and development in the relevant industries.

#### 2.3.5. Handheld Centrifugal Microfluidic Devices

Human blood analysis yields important health information. The analysis of serum biomarkers is an essential tool for modern medical diagnosis and bio information extraction [77]. To prepare blood samples, centrifugation is the first critical step and because an excess of red blood cells affects the background fluorescence and interferes with such detection [78], the plasma, serum, and parasites must first be separated [79]. Commercial centrifuges are expensive, bulky, require supporting equipment, such as power systems, and require trained personnel to carry out regular maintenance and inspections. Thus, conventional centrifuges are unsuitable for POC diagnosis in remote areas, and portable centrifuging equipment that can be used in resource-limited environments must, therefore, be developed [80].

**Figure 4:**
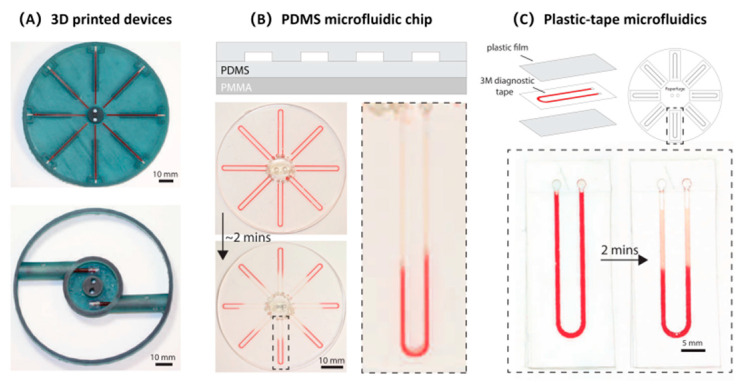
The classifications of handheld microfluidic centrifuge. (**A**) Handheld microfluidic centrifuge made with 3D printing technology. (**B**) PDMS handheld microfluidic centrifuge. (**C**) Plastic handheld microfluidic centrifuge. Reprinted with permission from [81]. Copyright 2017 Springer Nature.

Bhamla et al., developed an ultra-low-cost manual centrifuge called “paperfuge”. The cost of each centrifuge is no more than USD 0.20, and the device is very small with a weight of only 2 g. It can reach speeds of 125,000 rpm for 30,000× *g* of centrifugal force; thus, it is can easily separate whole blood. Centrifugal microfluidic devices can be developed by using 3D printing technology, PDMS, and plastic. Figure 4 represents the classifications of handheld microfluidic centrifuge [81]. These devices can be quickly put to use in resource-poor environments, providing a new direction of development for microfluidic POCTs.

**Figure 5:**
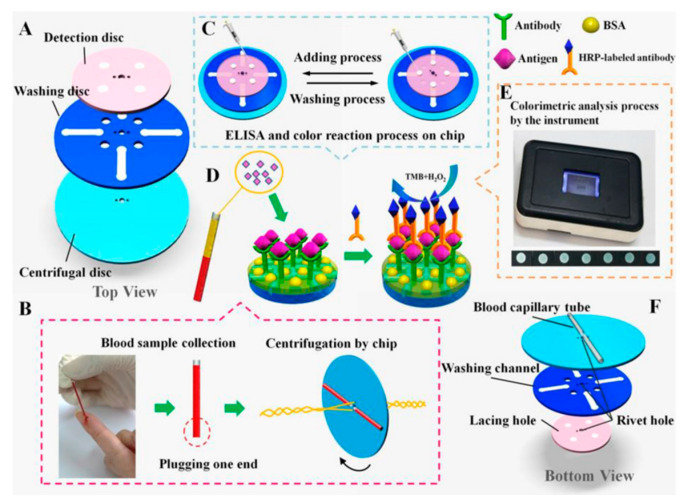
Structure and operation of FICA-μPADs. (**A**) Decomposition of a FICA-μPAD. (**B**) Blood samples from a finger puncture were placed into a capillary tube and centrifuged. (**C**–**E**) ELISA generates signals using a portable inter-reader. (**F**) Base image of the FICA-μPad, highlighting immune response areas, washing channels, and vascularization vessels. Reprinted with permission from [82]. Copyright 2020 Elsevier.

The paperfuge has some shortcomings. For example, it can centrifuge whole blood but it cannot effectively collect relevant samples. Li et al., improved the system and designed a paper-based integrated diagnosis system. The system effectively integrates the paperfuge and an immunoassay unit that can analyze biomarkers in blood while centrifuging. ELISA-level analysis of carcinoembryonic antigens (CEA) and alpha fetoproteins (AFP) in human serum was achieved. The system is called the ‘Fully Integrated hand-powered Centrifuge and Analysis paper-based microfluidic devices’ (FICA-μPADs). The operation of this system is also very simple. It only needs to settle the blood sample of the patient’s finger in the capillary tube of the hand-powered centrifuge for centrifugation, and then complete the corresponding ELISA operation through the immunoassay unit, before finally reading the corresponding signal through the portable inter-reader to obtain the experimental results. Crucially, in the absence of any process optimization, the manufacturing cost of a single unit was less than USD 0.50, which is suitable for POC detection. Figure 5 depicts the structure and operation of the FICA-μPADs [82].

**Figure 6:**
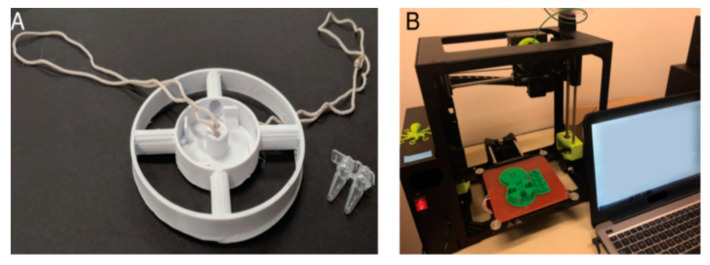
The production of 3D-Fuge. (**A**) Physical picture of a 3D-Fuge. (**B**) Printing of a 3D-Fuge. Reprinted with permission from [84]. Copyright 2019 Public Library of Science.

The development of 3D printing technology has resulted in new methods of rapid POC detection [83]. Byagathvalli et al., designed the 3D-Fuge using 3D printing technology, and it could process samples up to 2 mL, overcoming the limitation of traditional handheld centrifuges can only process 20 μL samples each time. Up to four samples could be stored and centrifuged simultaneously to achieve nucleotide extraction. The production cost of these devices is less than USD 1.00, and they can be produced with any type of 3D printing equipment. Thus, this design is suitable for large-scale production [84]. Figure 6 represents the production of the 3D-Fuge.

Since Bhamla et al., invented an ultra-low-cost manual centrifuge, the development of similar devices has accelerated. PDMS, plastic, and 3D-printed microfluidic centrifuges have been developed, broadening the potential applications of microfluidic POCTs.

However, these handheld centrifuges still have the inherent drawbacks of small-capacity systems. Additionally, many people doubt whether small, low-cost equipment can perform effectively. The market for and profit margin of handheld centrifuges is also low, limiting the development; however, these devices could be used in areas with limited resources and become more popular with time [85].

#### 2.3.6. Microfluidic POC Devices Using DEP Technology

Dielectrophoresis (DEP) is a label free, noninvasive, independent, rapid and sensitive particle manipulation and characterization technology. In the past decades, the progress of MEMS technology has made the biomedical application of DEP possible. The system developed through these technologies can use a small number of biological samples and reagents for analysis and detection. Lab on chip (LOC) equipment capable of processing micro/nano samples has promoted the development of biotechnology and chemistry.

DEP is a major electrical phenomenon in electrical technology. DEP can deal with charged particles and dielectric particles at the same time, such as cells and bacteria. DEP is a phenomenon of relative motion between suspension and medium under a non-uniform electric field. The force leading to this motion is called a dielectrophoretic force [86]. Designing and analyzing new and more advanced LOC equipment require accurate modeling and simulation of sample/particle dynamics in such devices. Through dielectrophoretic force, we can realize the control of small particles, so as to realize more possibilities [87].

Blood is mainly composed of plasma and cells, and there are a variety of cell types in blood. Therefore, blood contains significant information about systemic function. Moreover, the dielectric properties of cells can be separated by Dep. Han et al., designed a laterally driven continuous dielectric electrophoresis (DEP) differentiator, which can effectively separate red blood cells and white blood cells in whole blood samples [88]. In addition, DEP has many applications, including the separation of fetal cells from maternal blood, microbial separation, and stem cell and cancer cell separation [89].

POC detection equipment has been widely used in clinical laboratories and in patients’ disease detection. The development of POC equipment can provide a faster and low-cost method for disease diagnosis. With the development of micro manufacturing technology, cell separation and concentration based on DEP have become possible in practice and because DEP is simple to operate, does not need skilled technicians, has low voltage requirements and does not need high use requirements, DEP has the potential to be used in portable POC medical devices.

Chen et al., designed an integrated microfluidic platform that uses optically induced dielectrophoresis to separate and recover extracellular vesicles, which has high potential in cancer-related exosomal protein and miRNA analysis applications [90]. Sahin et al., proposed a microfluidic device which can use the DEP method to separate red blood cells and bacterial cells [91].

Although DEP technology and microfluidic technology have many applications, there are still many challenges to realize a POC application. Firstly, the accurate measurement of the dielectric performance of the battery is an important problem in the design of microfluidic POC equipment based on DEP. Secondly, the differences of the cell molecules and physiological states will also lead to changes of the dielectric properties, which leaves many applications in the experimental stage only and which cannot be put into use. Although there are still some problems to be solved, DEP technology can undoubtedly play an important role in microfluidic POC equipment, so that the microfluidic POC equipment has a wider range of applications. As these problems are overcome in the future, DEP technology can be used to manufacture many microfluidic POC devices for the early diagnosis and prognosis of diseases. Table 1 describes the overview of the microfluidic POC equipment types.

### 2.4. Advantages over Non-Microfluidic POC Devices

POCT can provide early and rapid diagnosis results for patients who need to detect related diseases. The advantages of POCT lies in its portability and rapid detection ability. POC equipment has formed a large commercial scale and has been applied in different disease detection fields [93].

It can be seen that there are many similarities between the microfluidic system and POCT. In fact, the microfluidic system has changed many applications of POCT in medical diagnosis. A microfluidic system has a high sensitivity in detection and can quickly obtain detection results, therefore, the combination of a microfluidic system and POCT could be used to design more portable and low-cost devices for rapid detection. The integration of microfluidics in medical point detection has significantly changed disease diagnosis and pathogen detection. Being easy to use, having no need for skilled personnel or heavy equipment, requiring a low sample size and delivering fast results makes POCT equipment an indispensable part of the healthcare industry.

There are many on-site detection and diagnosis POC devices in the market, including but not limited to glucose detection and infectious disease monitoring; however, among the POC detection devices, microfluidic POC devices with POC integrated microfluidic technology occupy the vast majority of the share, because microfluidic POC devices can allow fluid operation and detection to be carried out in the same device, which is more integrated than non-microfluidic POC devices. At the same time, many mature application technologies in the laboratory, such as ELISA and lamp, have been successfully applied to microfluidic devices, which greatly increases the disease detection ability of microfluidic POC devices. At the same time, there are many kinds of microfluidic POC devices, which can adapt to different detection environments, thus, it is easier to commercialize. Table 2 summarizes the advantages of microfluidic POC devices over non-microfluidic POC devices.

## 3. Microfluidic POC in Early Diagnosis of Infectious Diseases

### 3.1. Introduction

COVID-19 has dominated the recent discussion on infectious diseases [100]; it is a disease that has wrecked economies, strained healthcare systems, and caused widespread death, and many countries worldwide have had difficulty containing it [101]. Compared with other infectious diseases caused by coronaviruses such as the Middle East Respiratory Syndrome and Severe Acute Respiratory Syndrome (SARS), COVID-19 has a higher transmission rate and is more harmful [102]. The disease causes viral pneumonia; patients have a fever, cough, and other respiratory symptoms. The inaccurate diagnosis of COVID-19 may also lead to rapid community spread.

Many infectious diseases have affected humans, including the bubonic plague in the middle ages [103], influenza A (H1N1) in the early 20th century [104], SARS [105], acquired immune deficiency syndrome (AIDS) [106], and tuberculosis [107]. These diseases can be transmitted through many vectors. In particular, respiratory transmission is rapid, causing the large-scale spread of a virus and complicating the control of the infectious disease. Modifying human behavior can facilitate the control of infectious diseases. For example, condoms can effectively reduce the spread of AIDS, and avoiding gatherings of people can reduce the spread of respiratory infectious diseases. Early diagnosis is also effective for reducing transmission at the beginning of an outbreak. The early diagnosis of COVID-19 is a key measure for controlling its spread and providing timely treatment to infected people. Therefore, rapid and accurate detection is critical for an effective public health response.

Virological and serological diagnostic techniques are the primary methods for early COVID-19 screening [108]. Serological detection methods include ELISA, colloidal gold immunochromatographic assay, chemiluminescence immunoassay (CLIA), and real-time reverse transcription-polymerase chain reaction (RT-PCR) [109]. The high cost of both RT-PCR and ELISA may limit their use in underdeveloped regions. Therefore, fast, cheap, and easy-to-use early diagnostic tools for COVID-19 must be developed to effectively isolate infected people and avoid further spread of the disease. POCTs constitute a forward-looking approach for solving this problem.

Compared with their conventional counterparts, microfluidic devices can more accurately and more conveniently quantify antibodies and biomarkers. Microfluidic devices can be combined with traditional detection methods to achieve rapid and efficient POC detection of the SARS-CoV-2 virus. Therefore, microfluidics have excellent potential for SARS-CoV-2 virus detection and could be commercialized.

### 3.2. Application of Infectious Disease Detection

Immunoglobulin M (IgM) is the first line of defense against the invasion of viral pathogens, and Immunoglobulin G (IgG) antibodies are produced at a later stage and cause long-term immunity. Therefore, the development of and recovery from diseases can be determined by detecting antibodies.

Lin et al., designed a detection tool integrating a diagnostic microchip, domestic portable fluorescent detectors, and a microfluidic immunoassay that could detect IgG, IgM, and antigens, improving the sensitivity and accuracy of SARS-CoV-2 diagnosis [44].

Certainly microfluidic chips still have shortcomings in detecting the SARS-CoV-2 virus and most microfluidic chips used for SARS-CoV-2 antibody detection still use bead-based sandwich-type immune assays, requiring long incubations and detection times. Therefore, fast detection of SARS-CoV-2 antibodies by using a microfluidic chip still needs further development [110].

If collection of nasopharyngeal specimens is impossible, saliva can be used as a test specimen [111]. The use of saliva samples provides various advantages, for example, patients can collect the samples themselves, thus reducing opportunities for viral transmission within crowds. In fact, the method of using human saliva as a sample to detect the virus has had many successful applications. Davidson et al., designed a paper-based microfluidic POC detection device to detect SARS-CoV-2 in saliva by loop-mediated isothermal amplification [112]. Wang et al., designed a paper-based microfluidic POC device using reverse-transcription loop-mediated isothermal amplification (RT-LAMP) to detect SARS-CoV-2 in saliva. The device only needs a simple heat source to carry out the experiment it does not need to extract and concentrate RNA in advance, and the results can be obtained by observing the colorimetric response with the naked eye. Therefore, it is very suitable for areas with limited resources [113]. Farshidfar formulated a smartphone-based microfluidic system for the detection of the COVID-19 virus in saliva that facilitates the early diagnosis of COVID-19 [114].

AIDS is another common and incurable infectious disease. AIDS is the stage of a human immunodeficiency virus (HIV) infection in which the level of CD4 + T lymphocytes is lower than the critical level. Approximately 37 million people are estimated to have HIV globally [115] and according to figures released by the WHO in 2019, AIDS claimed 770,000 lives in 2018. As many as 20% of individuals with HIV do not know that they have been infected, facilitating the spread of the virus [116]. If AIDS patients can take their medication in the correct manner, they can control their disease and significantly reduce the probability of transmitting HIV to others. Therefore, HIV detection is also critical for public health [117]. Yang et al., developed a microfluidic immunoassay box with a handheld optical reader that uses patient urine to test for HIV. The total cost is only USD 70, and test results can be provided in a few seconds. Therefore, this test is suitable for use in developing countries with poor infrastructure [118].

Tuberculosis is a fatal infectious disease that can spread rapidly through respiration. A 2019 report stated that 1.4 million people had died of the disease worldwide that year [119]. The incidence and mortality rates of tuberculosis are increasing in Africa and Southeast Asia. In 2020, Mbano et al., used an efficient, simple microfluidic platform and the simple linear workflow of microfluidic high-resolution melting analysis (HRMA) to create Light Forge, a low-cost tuberculosis test for resource-limited environments. The experimental results revealed that the results of this method were consistent with conventional detection results; thus, Light Forge is a promising prototype of a rapid and inexpensive tuberculosis diagnosis test suitable for resource-limited environments [120]. Table 3 depicts the overview of applications of Microfluidic POC devices in the early diagnosis of infectious diseases.

### 3.3. Prospects for the Detection of Infectious Diseases

Microfluidic technology already has many applications in the field of infectious diseases. The importance of telemedicine technologies has been widely recognized during the COVID-19 pandemic, which has transformed the lifestyles of many people [121]. A disease database concerning patients should be established in a timely manner to aid the pandemic response. Statistical and mathematical models or artificial intelligence methods could be used to understand the outbreak modes of infectious diseases to identify appropriate solutions for the next infectious disease outbreak [122,123].

**Figure 7:**
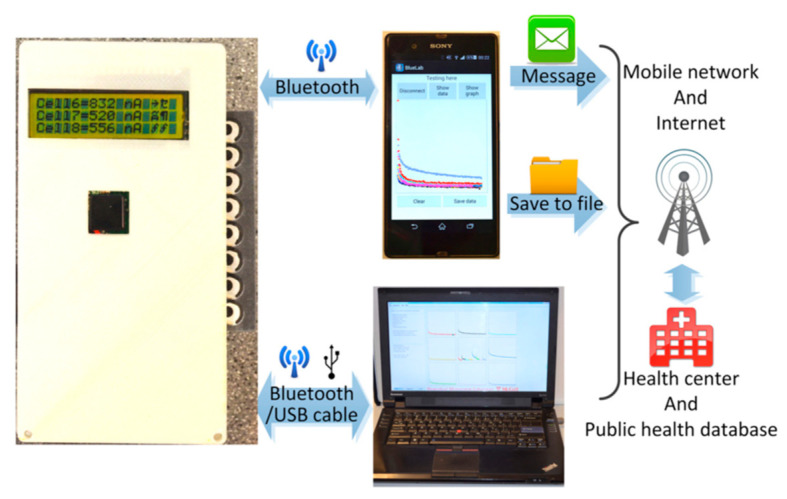
Paper-based microfluidic platform with wireless communication for telemedicine. Reprinted with permission from [127]. Copyright 2016 AIP Publishing.

Many applications of telemedicine have been realized during the pandemic. Remote health monitoring can be performed with wearable sensors and smartphones. Commercial wearable sensors can collect patient physiological parameters (e.g., heart rate, respiratory rate, pulse rate, sleep time, and body temperature) and transmit them to a smartphone for analysis and processing. These wearable devices can also continuously transmit biological data to the internet in real time, so as to convert this information into clinical knowledge for monitoring the physical condition of patients [124]. Thus, anomalies in a patient’s physical condition can be identified early for effective isolation before the onset of COVID-19 [125]. New technologies, such as high-resolution cameras, encryption software, electronic stethoscopes, microfluidic diagnostic systems, and broadband internet have expanded the potential of telemedicine [126]. Zhao et al., designed a paper-based microfluidic platform to detect HIV and hepatitis C virus antibodies in serum. The platform has a wireless communication module for telemedicine, which is shown in Figure 7 [127], while wearable sensors are also essential for clinical diagnosis and patient monitoring [128].

Traditional immune monitoring requires trained personnel and specialized equipment. With the advent of microfluidic chips that can be used to perform immune detection, the rapid detection of COVID-19 can be achieved without highly skilled professionals and tools. Through artificial intelligence, Internet of Medical Things technologies, and an appropriate immunosensor, doctors can conduct timely diagnoses and communicate with the patient through telemedicine platforms [129]. Similarly, 5G wireless transmission technologies have improved telemedicine services. Providing high speeds at 10 Gbps with low latency and wide coverage, 5G has increased the potential of telemedicine services. As 5G networks become established in less-developed areas, telemedicine can be performed in these areas, facilitating the rational allocation of medical resources; however, widespread deployment of 5G technology still requires additional time [130,131].

These emerging detection systems can be tested during the current pandemic to determine whether they are effective detection solutions. COVID-19 is unlikely to be the last pandemic to affect humanity. Therefore, existing technologies must be continually improved and expanded in preparation for future public health crises.

## 4. Microfluidic POC in Early Diagnosis of CVDs

### 4.1. Introduction

According to the WHO, the leading cause of death worldwide is CVDs, causing 17.9 million deaths annually. Over 75% of these deaths occur in underdeveloped areas [132,133,134]. High rates of CVDs can be attributed to rapid lifestyle changes and increased life expectancy in developing countries, but these rates also indicate that medical resources in developing countries are still inadequate compared with those in developed countries [135]. These non-communicable diseases also constitute a public health crisis in developing countries.

The cardiovascular system consists of three parts: the heart, the blood, and the blood vessels [136]. The heart generates pressure to circulate blood throughout the body [137]. CVD, also known as heart disease, refers to diseases of the heart and blood vessels, including atherosclerosis and hypertension. The most common CVDs are coronary heart disease (CHD) and cerebrovascular disease (stroke). CHD and stroke are typically caused by atherosclerosis. Many other types of CVD exist, including heart disease caused by hypertension and inflammatory heart disease [138].

In the past decade, biomarkers have been used in various clinical applications, including detecting CVDs and numerous CVD biomarkers have been discovered. Early diagnosis can reduce mortality and total treatment costs for patients with CVDs [139], and POC devices can detect CVDs early by detecting CVD biomarkers.

### 4.2. Detection of Multiple Biomarkers for CVD

The use of a single biomarker to diagnose CVD has some limitations, for example, a single biomarker may be associated with multiple diseases and may provide an incorrect diagnosis. Therefore, the simultaneous detection of numerous CVD biomarkers is more reliable, improving specificity, reducing analysis time and cost, and more quickly identifying the risks of CVD [140]. Research platforms that can simultaneously detect multiple cardiac biomarkers have been developed [141]. Clinicians advocate the simultaneous detection of multiple cardiac biomarkers for the proper evaluation of CVDs as multiple analyses provide more comprehensive, effective, and accurate diagnostic information, saving time, reagents, and labor costs. Therefore, the numerous analyses of biomarkers are more advantageous than the measurement of individual biomarkers [142].

Microfluidic-based organ chips can facilitate research on the pathogenesis of CVDs for developing diagnostic and treatment methods [143] and many microfluidic POC devices that can detect multiple biomarkers are in use today.

**Figure 8:**
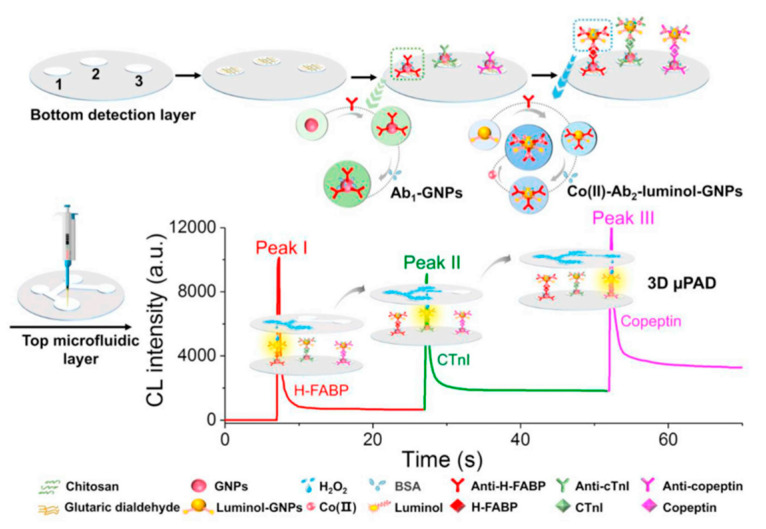
Schematic of 3D μPAD with three detection zone for multiple immunoassays of H-FABP, cTnI, and copeptin. Reprinted with permission from [146]. Copyright 2020 Elsevier.

AMI is one of the most dangerous CVDs. AMI is a life-threatening disease and one of the most common causes of death. The simultaneous determination of multiple biomarkers of AMI is key to the accurate detection of AMI. Many biomarkers have been identified for AMI diagnosis, including troponin I (cTnI), H-FABP, and copeptin. cTnI is the gold standard biomarker for the early diagnosis of AMI [144,145]. Li et al., developed a 3D microfluidic paper analysis device (μPAD) with multiple analysis capabilities which has three detection zone. The device uses temporally resolved chemiluminescence (CL) emissions to measure H-FABP, cTnI, and copeptin simultaneously. The device has great potential for early AMI diagnosis and Figure 8 represents the schematic of a 3D μPAD [146].

Boonkaew et al., developed a paper-based microfluidic that can simultaneously measure the levels of three essential CVD biomarkers: C-reactive protein (CRP), cTnI, and procalcitonin (PCT). The method features multiple detection regions and multiple working electrodes that can simultaneously detect the three CVD biomarkers in a single sample with high selectivity and sensitivity [147]. Sinha et al., designed an integrated microfluidic POC system with a field-effect transistor (FET) sensor array that could detect a variety of cardiac biomarkers simultaneously. Four biomarkers of CVD, including CRP, N-terminal pro b-type natriuretic peptide (NT-proB NP), cTnl, and fibrinogen, could be detected in clinical samples (approximately 4 µL) within 5 min [148]. Table 4 depicts the overview of applications of microfluidic POC devices in early diagnosis of cardiovascular diseases.

### 4.3. Prospects for the Detection of CVDs

Future research may focus on various aspects such as the use of saliva samples, wearable devices, telemedicine, and fluorescent coded microsphere technology for the detection of vascular diseases.

Most protein biomarkers produced by the human body are released into the blood, urine, or other fluids and thus can be detected in these fluids [149]; however, the detection of protein biomarkers in medical testing typically uses blood samples. Recently, protein biomarkers that can detect CVDs have been discovered in urine and saliva. Urine and saliva sampling is not invasive, and these samples are not easily disturbed but are easy to collect. Thus, they are ideal choices for rapid POC detection. Saliva can be used for the noninvasive diagnosis of oral and systemic diseases; therefore, saliva is expected to replace blood. Saliva is a complex liquid containing enzymes, electrolytes, proteins, nucleic acids, antibacterial components, hormones, cytokines, and antibodies. Saliva reflects the health status and diseases of the human body and analysis of the physical condition of the human body using saliva may be a goal of future medical research [150]. Saliva contains numerous CVD-related biomarkers, including Myoglobin (MYO), cTnl, and creatine phosphokinase MB (CK-MB). Therefore, human saliva may be collected for future POC CVD detection tests [151].

Wearable devices may also become common in the POC detection of CVDs. Ramasamy et al., designed a sensor system that can be worn on the body. The system incorporates intelligent wireless communication technology and can be used by doctors to make diagnoses using, for example, the wearer’s electrocardiogram (ECG) and electroencephalogram (EEG) readings. This information could be used by doctors to judge the condition of the patient’s heart and nervous system for the early detection of CVD and determination of the neurological system disease risk [152].

Telemedicine is another potential direction for research on future POC technologies. Vishwanatham et al., developed a portable POC device that could be used for the ECG monitoring of rural residents. The device is composed of three modules. First, a hardware module is used to collect ECG data and transmit it to a mobile phone wirelessly via Bluetooth Low Energy, which is then used to analyze and present the received data. Finally, the information is sent to a remote server, enabling doctors to view the patient’s electronic health records and leave comments or suggestions for the patient [153].

The rapid and simultaneous detection of DNA and protein biomarkers is also critical for the detection and monitoring of CVDs. Dinter et al., developed a microfluidic technology platform that enables the simultaneous detection of CRP, brain natriuretic peptide, and low-density lipoprotein. The platform uses fluorescent coding microsphere technology and the open-source tool *digilogger* to analyze and process the data. The method achieved faster detection speeds and higher detection accuracy than conventional immunoassay methods; however, the platform still requires supporting equipment for analysis, which may not be available in underdeveloped areas. We hope that this high-precision equipment could be miniaturized and produced inexpensively to allow for application in regions with weaker infrastructure [154].

## 5. Microfluidic POC in Early Diagnosis of Tumors

### 5.1. Introduction

Cancer is a complex disease that is difficult to treat and classify, and it is a significant disease affecting human health [155]. The incidence and mortality rates of cancer are increasing worldwide. In 2018, new cancer cases numbered 18.1 million, and deaths due to cancer reached 9.6 million. Lung cancer, breast cancer, colorectal cancer, prostate cancer, gastric cancer, and liver cancer are the most common types of cancers [156].

The early diagnosis of cancer is critical for improving clinical efficacy; however, the symptoms of early-stage cancer are rarely recognized, leading to a delay in treatment [157]. Late-stage diagnosis significantly reduces patient survival rates. The conventional methods for early cancer diagnosis include imaging and serology while the imaging methods include ultrasound, computed tomography, and magnetic resonance imaging. Because of the expense and radiation exposure entailed by the imaging monitoring methods, they are unsuitable for routine examinations.

Current cancer diagnosis methods typically use hematology tests. Screening for cancer with serum markers can reduce medical costs and harm to patients and is therefore widely used for the detection of early-stage cancer [158]. Moreover, the measurements of protein biomarkers are important indicators for early cancer screening and cancer monitoring and treatment [159].

### 5.2. Applications for Cancer Detection

Many successful applications of microfluidic devices for cancer detection have been reported. The DNA methylation of tumor suppressor genes has been recognized as a diagnostic biomarker for early tumorigenesis. Wang et al., designed an integrated microfluidic system that can perform the entire process of DNA methylation analysis. The entire process, from sample loading to result from analysis, takes only 3 h, realizing rapid diagnosis of early cancer [157]. The method is simpler and faster than the conventional, standard detection method using a sodium bisulfite treatment and *HpaII/MspI* endonuclease.

CA-125 is an important biomarker of cancers and concentrations of CA-125 can be used to monitor the progress of cancer. Nunna et al., designed a POC system combining a biochip and a microfluidic that could monitor the progress of cancer in real-time by measuring the CA-125 concentration in blood from a finger puncture [160].

Lung cancer (LC) is the leading cause of cancer-related death worldwide, with a high incidence rate and mortality rate. Among them, non-small cell lung cancer (NSCLS) is the main case type of lung cancer, accounting for more than 80% of all cases [161]. Exosomes have become a new biomarker for the early treatment and detection of lung cancer. Yang et al., proposed an integrated microfluidic device, which can adjust the appropriate membrane pore size by ion sputtering gold layers with a different thickness, so as to realize the isolation of foreign bodies and body fluids through the pore size, to therefore separate the specific exosomes of lung cancer from the urine of patients. As a result, the method is very promising for distinguishing patients with early lung cancer from healthy individuals [162]. Microfluidic POC devices can also detect cancer biomarkers for other cancer types. Prostate cancer (PCa) is the most common cancer in men in western countries. Europe is estimated to have had 450,000 new cancer cases and 107,000 cancer deaths annually from 2018 [163]. Prostate-specific antigen (PSA) is still the most commonly used biomarker for the early detection of Pca [164]. The concentration of PSA in the serum of healthy men is in the range of 0–4 ng/mL; this concentration increases in patients with prostate cancer. Therefore, the PSA index is critical for the detection of prostate cancer, and individuals with PSA levels in the range of 4–10 ng/mL are very likely to develop cancer. Therefore, if a device could quickly and cost-effectively detect PSA indices lower than 4 ng/mL, the diagnosis of prostate cancer patients could be improved [165]. Mandal et al., designed a system integrating dielectrophoresis (DEP), graphene FETs, and a compact disc-based microfluidic to detect PSA content of less than 4 ng/mL in blood serum, thus this could be used for the early screening for prostate cancer [166].

The most common types of cancer in women are breast cancer and cervical cancer. Cervical cancer is the most common cancer in women and is caused by the human papillomavirus (HPV) [167]. The early monitoring of cervical cancer is necessary because early cervical cancer can be completely cured. Karakaya et al., developed a paper-based microfluidic chip that achieved the early detection of cervical cancer by measuring HPV 16 and HPV 18. The detection could be completed in less than 40 min [168]. Lim et al., developed a system that integrates a microfluidic chip, 3D-nanostructured hydrogels and exosomatic mRNA sensors. The system can further detect the exosomal ERBB2 in the blood associated with breast cancer, thus proving the validity of the system in the diagnosis of breast cancer [169]. Table 5 depicts the overview of applications of microfluidic POC devices in the early diagnosis of tumors (cancer).

### 5.3. Prospects for the Detection of Cancer

Many possibilities exist for future uses of microfluidics for cancer detection including POC applications of exosomes, multiple cancer biomarker POC detection, tumor cell cultures, telemedicine, and anti-cancer drug screening on microfluidic chips.

Due to the abundance of nucleic acids and proteins, exosomes are the cutting-edge biomarkers for cancer diagnosis. Although exosomes have potential as tools for cancer detection and monitoring, they are small and of low density; therefore, they are time-consuming and expensive methods, such as those featuring ultracentrifugation, immunomagnetic beads, or commercial kits that are required for analysis. Recently developed microfluidic platforms can effectively separate and detect exosomes from liquid biopsy and can achieve a higher sensitivity compared with conventional methods, enabling the detection of exosomes in future POC applications [170].

The detection of multiple disease biomarkers with high sensitivity and rapidity is critical for tiny liquid samples. Chen et al., introduced a microfluidic platform combining magnetic-based single bead trapping and acoustic micromixing that could detect many tumor biomarkers within minutes. The system used mathematical models to simulate real fluid control conditions, and Chen et al., demonstrated the platform’s ability to quickly detect biomarkers, thus verifying the platform’s ability to quickly detect multiple cancer biomarkers in future applications for POC diagnosis [171].

Microfluidic technology can simulate a tumor microenvironment on a chip to cultivate tumor cells and screen anti-cancer drugs on the chip, saving companies hundreds of millions of dollars in research and development funding. This is another application of microfluidic technology in cancer diagnosis [172].

Although microfluidic chips are imperfect for cancer detection and diagnosis, the development and optimization of microfluidic technology are ongoing. The development of microfluidic devices for cancer detection requires close cooperation between biomedical and clinical researchers, while some existing detection technologies could be integrated into microfluidic chips, increasing the applications of microfluidic technology in cancer diagnosis. The future development of microfluidic chips is expected to continue to decrease their size and cost; therefore, more products for POC applications could soon be produced for the detection and treatment of cancer.

## 6. Microfluidic POC in Early Diagnosis of Chronic Diseases

### 6.1. Introduction

Poverty is strongly related to chronic disease. Due to the lack of timely medical services in underdeveloped areas, some diseases may develop into chronic diseases, or existing chronic diseases may be left undiagnosed or not be subject to proper treatment [173]. According to the WHO, 80% of deaths caused by global chronic diseases occur in low- and middle-income countries [174].

Chronic diseases include all health problems that must be continually managed for at least six months. These diseases have a long duration, progress slowly, and do not spread from person to person. Patients with chronic diseases tend to require long-term supervision, observation, or care [175] and chronic diseases (e.g., CVD, diabetes, stroke, lung disease, and kidney disease) are often difficult to cure. The treatment difficulty only increases as the disease progresses, making the timely detection of these diseases critical [176].

### 6.2. Application of Chronic Disease Detection

Anemia is a common chronic disease and iron deficiency anemia is the most common type. Anemia is a disease in which the concentration of hemoglobin (Hb) or the number of red blood cells in the human body is lower than normal [177]. Anemia can cause physical weakness in patients, leading to increased incidence and mortality rates, lower productivity, and impaired neurodevelopment. Anemia, as a common chronic disease, affects one-third of the world’s population [178]. Anemia can be diagnosed by detecting Hb levels in a patient’s blood sample. Hematology analyzers are used in conventional laboratories to detect Hb concentrations, but these devices are not portable.

Taparia et al., designed a microfluidic system that uses optical methods to detect Hb levels in whole blood for anemia detection [179], while Plevniak et al., designed a microfluidic POC system called iPOC (3D) for anemia diagnosis. The device could be integrated with smartphones to perform rapid tests through colorimetric measurements. The total cost per test was only USD 0.50, demonstrating the method’s suitability for use in resource-limited regions or low-income countries [180].

Chronic heart disease and chronic kidney disease are two other common chronic diseases; CRP is an essential biomarker of both diseases. Because special laboratories are required to detect these chronic diseases, POC results cannot be seen rapidly or in real-time. To solve this problem, Dong et al., developed a system integrating a paper-based microfluidic immunoassay with a smartphone for CRP detection in the blood. The detection was rapid and inexpensive and thus could be used to improve the prognosis of chronic heart and kidney disease [181]. For patients with advanced chronic kidney disease (CKD) and patients with kidney damage who need continuous dialysis treatment, the concentration of phosphate in their blood plays an important role in their disease monitoring. Ray et al., designed a paper-based microfluidic POC device, which can accurately measure the phosphate concentration in the patient’s serum in about 45 min by using the smartphone and the corresponding 3D-printed smartphone attachment. The 3D-printed smartphone attachment weighs only 400 g and costs no more than USD 80. In the case of a small batch disposable use, the cost does not exceed USD 3.50. Therefore, it is very suitable for patients who need to monitor serum phosphate levels frequently [182].

Diabetes is another complex chronic disease; diabetes is a metabolic disorder that manifests as hyperglycemia caused by insufficient insulin secretion. A lifetime of insulin injections is required to control blood sugar. Glycated Hb (HBA1C) is an essential diagnostic tool for detecting and managing patient conditions [183].

HBA1C is an essential parameter for indicating blood glucose levels and the monitoring of blood glucose levels is helpful for patients with diabetes, and can protect their internal organs [184]. Chang et al., developed an integrated microfluidic system for measuring glycosylated hemoglobin. The system uses nucleic-acid aptamers to achieve high sensitivity and high specificity detection of HBA1C. The system can return results within 25 min and is lower the cost than traditional methods, thereby minimizing the risk of diabetic complications [185]. Glucose is also an important marker for the detection of diabetes. The general paper-based microfluidic devices are easily affected by the sample volume. Choobbari et al., designed a paper-based microfluidic device that only related to the glucose concentration in the sample without accurately controlling the sample volume and the corresponding detection results could be obtained by analyzing the images captured by a smartphone [186]. Although blood is the most commonly used biological fluid for clinical diagnosis, some patients feel pain, fear, or discomfort when blood is drawn [187]. Saliva contains glucose, a marker for monitoring diabetes, and can be collected painlessly; thus, saliva is preferable to blood for diabetes detection [188]. Castro et al., designed a paper-based microfluidic device that used a colorimetric method to detect glucose in saliva. The paper-based microfluidic device can be integrated into a 3D printing stand as a tooth protector to place into the user’s mouth, so as to achieve real-time monitoring of blood glucose in diabetics [189].

**Figure 9:**
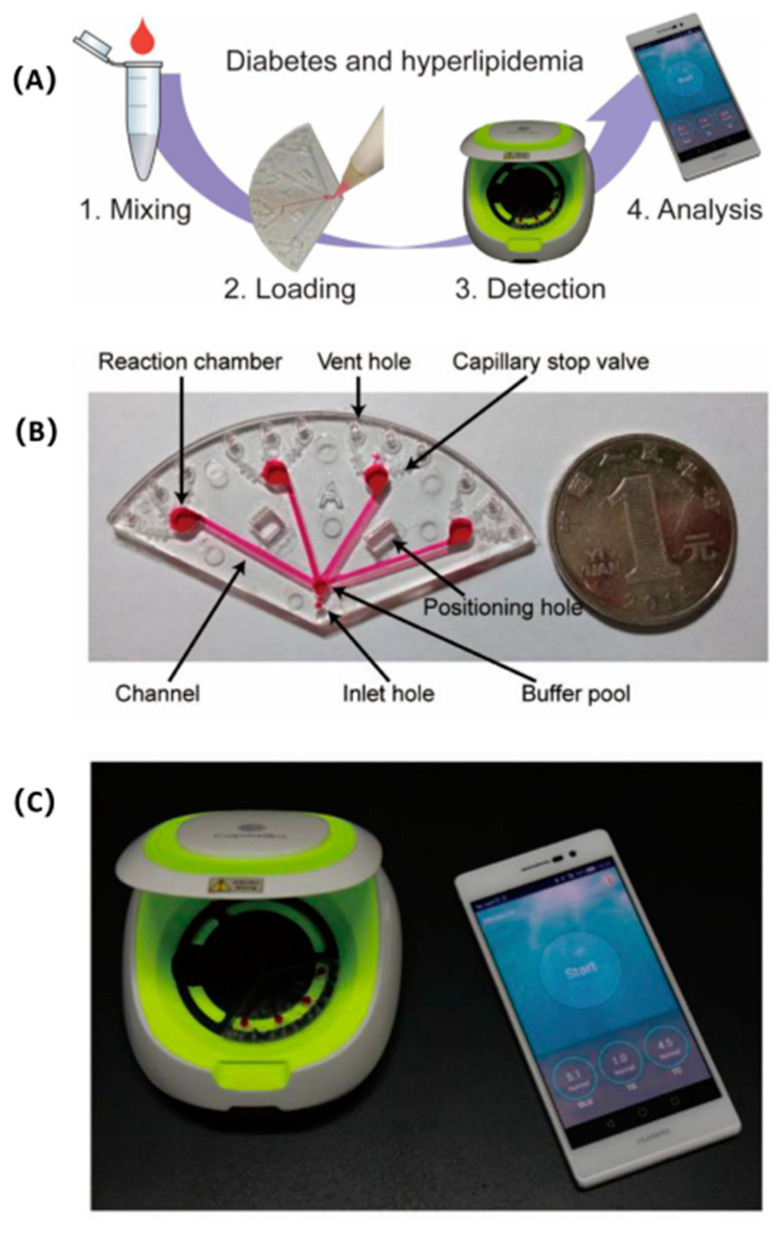
The process of the microfluidic chemical analyzer for analyzing diabetes and hyperlipidemia. (**A**) The process has four steps. First, the serum sample is mixed with a reagent. Second, the mixed solution is injected at the entrance of the microfluidic chip. Third, the detector is used for detection and analysis. Finally, the mobile phone is used to read the data. (**B**) Schematic of a microfluidic chip for multi-index analysis. (**C**) A microfluidic chemical analyzer assisted by a smartphone was used to analyze the color changes of two reagents in the chip reaction chamber. Reprinted with permission from [190]. Copyright 2019 Elsevier.

Diabetic patients usually exhibit one or more complications, with hyperlipidemia being the most serious. Hyperlipidemia is a common chronic disease that may lead to CVD. Therefore, the three indicators of glucose, triglycerides, and total cholesterol must be measured simultaneously to monitor diabetes and hyperlipidemia in real-time. Li et al., designed a smartphone-assisted microfluidic chemical analyzer that uses image-based colorimetric detection to simultaneously analyze these three indicators of diabetes and hyperlipidemia [190]. Figure 9 depicts the process of the microfluidic chemical analyzer for analyzing diabetes and hyperlipidemia. Table 6 depicts the overview of applications of microfluidic POC devices in the early diagnosis of chronic diseases.

### 6.3. Prospects for the Detection of Chronic Diseases

Future applications of microfluidic technology in chronic disease detection could focus on portable detection equipment, microfluidic organ chips, and more advanced microfluidic POC detection equipment.

Conventional medical systems must transform to account for an aging and larger population with a higher prevalence of chronic diseases. Since the 20th century, wearable devices have been used in healthcare and regular disease monitoring [191]. Recently, μPADs and wearable sensors have exhibited great potential for clinical diagnosis [192]. Castro et al., integrated μPADs into masks as wearable sensors to measure glucose levels by collecting saliva samples to monitor diabetes in patients [189]. Self-examination devices for chronic diseases could also be developed in the future. Zhu et al., proposed a microfluidic analyzer that could be used for the biochemical analysis of blood. Users can easily detect three commonly used indicators of diabetes and hyperlipidemia, including blood sugar, total cholesterol, and triglyceride, and can receive results in 15 min [193].

The development of microfluidic chips as organ models is also a direction for research and treatment of chronic diseases in the future. Approximately 10% of people in the world have chronic kidney disease, and that proportion is increasing; therefore, new methods are required to prevent kidney disease from becoming more common. Bioprinting is a promising method that can print organ structures to study underlying pathological mechanisms. An in vitro model of a kidney can be designed using 3D microfluidic organ chips to study treatments for relevant diseases [194]. Pancreas-liver microfluidic organ chips have been developed to study the mechanism of Type 2 diabetes mellitus and to develop drugs and treatment methods [195].

Microscopes and flow cytometry are commonly used in laboratories, but these devices cannot yet be used routinely in underdeveloped areas. Microfluidic technologies could be used to develop POC urine detection devices by integrating a lensless imaging device to achieve low-cost image flow cytometry for detecting and monitoring patients with chronic diseases. For future regular disease detection, microfluidic chips may be combined with different technologies to achieve miniaturized, high-sensitivity devices that can be used in numerous POC applications [196].

## 7. Conclusions and Prospects

Medical detection in areas that lack resources is a cause for concern. Microfluidic POC detection equipment for medical detection is rapid, low-cost, and easy to use; thus, these devices are a potential solution to this problem.

Microfluidic devices are typically manufactured using PDMS, paper, or 3D printing technology. Platforms integrating smartphones with microfluidic equipment are often effective for microfluidic POC applications and handheld microfluidic centrifugal devices and microfluidic POC devices using DEP technology are another avenue for microfluidic POC detection. There are different types of microfluidic POC equipment, but in the final analysis, it can meet the low-cost real-time detection of different regional environments. Therefore, microfluidic POC equipment is often small in volume and weight, with low manufacturing difficulty and cost, while it can obtain detection results in a short time.

In medical laboratories, disease biomarkers are typically analyzed for disease detection. Microfluidics and the related technologies are inexpensive, portable, and have sensitive detection; therefore, this technology can meet the requirements for medical testing in remote areas. Microfluidic POC devices have been successfully applied in infectious diseases detection, CVD detection, tumor detection, and chronic disease detection And the detection of diseases by microfluidic POC devices is typically achieved through the detecting of biomarkers and antibodies in serum. Microfluidic devices can now combine many detection technologies, such as ELISA, RT-LAMP, DEP, mass spectrum, and SPR, with smart phones and different detection accessories, and the microfluidic devices have been able to detect biomarkers with a high accuracy and sensitivity, which meets the needs of disease detection in many cases. With the continuous combination of related technologies and microfluidic devices, microfluidic detection devices have also been successfully applied with a high specificity and very low detection limit. Notably, the continued progression of artificial intelligence, big data, 5G technology, wearable devices, and databases established through patient information and telemedicine services, has exhibited much promise.

Although microfluidic technology has many advantages, it also has shortcomings. The commercialization of microfluidics is subject to customer and market acceptance. Therefore, maximizing the profits from commercial microfluidic devices is a concern for investors; however, the microfluidic industry has been growing steadily in the past five years worldwide with the compound growth rate of microfluidics in the medical equipment market being 22%, indicating that the commercialization of microfluidic medical equipment is increasing. The number of microfluidic-based devices submitted to the U.S. Food and Drug Administration and other regulatory agencies is also steadily growing and although microfluidic devices have still not been completely accepted by the market, a strong demand already exists.

Microfluidic POC detection technologies have transformed the methods of disease diagnosis. Professional operators are not required, and the results can be obtained quickly with only a few samples; however, although these devices have existed for decades, most microfluidic chip systems have not been commercialized or used as laboratory-level research instruments. Current POCT diagnostic equipment is still in the early stages of development and cannot compete with complicated experimental processes. Many challenges must be overcome before POCT diagnostic devices become standard clinical equipment. The microfluidics community still lacks standards and guidelines; these are necessary for product development in any industry. This lack of industry standards may become an obstacle to the commercialization of microfluidic devices. Therefore, the microfluidic industry must formulate measures, and drawing on the successful experience in other fields is helpful toward this end. After standardization, the industries can become more efficient, and the time and cost of the development process can be reduced. Additionally, those in the microfluidic field should increase their investment in biomedical applications and attempt to increase the adoption of microfluidics in clinical trials.

## Figures and Tables

**Table 1:** The overview of microfluidic POC equipment types.

Microfluidic POC Equipment Types	Constituent	Manufacturing Method	Comment	Ref.
Microfluidic equipment made of PDMS	PDMS polymer	Soft lithography	PDMS and soft lithography technology are of great significance to microfluidic devices.	[54]
Paper-based microfluidic device	Paper	Patterning technology	Paper chips are fast and inexpensive analysis platforms for the early diagnosis and treatment of diseases.	[61]
3D-printed microfluidic device	Thermoplastic polymers (used for FDM) or photocurable resins (used for SLA)	3D printing technology	3D printing technology can make microfluidic devices faster and leverage experimental findings to realize commercial devices.	[69]
Mobile sensors based on integrated microfluidic devices and smartphones	Microfluidic chip, smart phone, external sensors	Integration of a mobile phone and microfluidic chip	Data and image processing capabilities of integrated system are key for POC detection.	[92]
Handheld centrifugal microfluidic device	PDMS, plastic, paper, and 3D-printed devices	Soft lithography or 3D printing technology	New opportunities for electricity-free POC diagnostics.	[81]
Microfluidic POC devices using DEP technology	PDMS polymer, DEP technology	Soft lithography	DEP technology has great potential in the applications of microfluidic POC devices.	[89]

**Table 2:** The advantages of microfluidic POC devices over non-microfluidic POC devices.

Attribute Value	The Advantages of Microfluidic POC Devices	Ref.
Integration	Microfluidic POC devices have better integration.	[94]
Compatibility	The mature technology of the laboratory is easy to migrate to the microfluidic POC devices.	[95,96]
Commerciality	There are many categories, which can adapt to more disease detection occasions. The design and manufacturing are more modular and easier to be put into use in large-scale manufacturing.	[97,98,99]

**Table 3:** The overview of applications of Microfluidic POC devices in early diagnosis of infectious diseases.

Classification	Detection Target	Composition	Sample	Comment	Ref.
ZIKV	Zika virus	3D-printed microfluidic device	Saliva	The device can achieve rapid detection of ZIKV.	[70]
		Wax paper microfluidic chip and smartphone	Serum	The color change of ZIKV RNA in the microfluidic detection area can be seen within 15 min, and virus detection can be completed with a smartphone camera.	[75]
COVID-19	IgG, IgM and antigens of SARS-CoV-2	Microfluidic system integrated with a diagnostic microchip and portable fluorescence detector	Serum or pharyngeal swabs	The assay had high sensitivity and specificity.	[44]
	SARS-CoV-2	Paper-based microfluidic device using LAMP	Saliva	This device can detect the virus in a short time and has high analytical sensitivity and specificity.	[112]
		Paper-based microfluidic device using RT-LAMP	Saliva	The device can be used as a supplement to current point-of-care and community testing procedures.	[113]
		Microfluidic system based on smartphone	Saliva	The system could have a substantial influence on the epidemiology of the disease.	[114]
AIDS	HIV	Microfluidic immunoassay box with a handheld optical reader	Urine	The system can provide more convenient, easier to operate, and more affordable HIV urine testing in POC diagnostics.	[118]
Tuberculosis	Tuberculosis virus	Microfluidic platform and linear workflow of HRMA	Mycobacteria tuberculosis Isolates	A promising prototype for a fast, low-cost diagnostic alternative for detection of drug resistant strains of tuberculosis in resource-constrained settings.	[120]

**Table 4:** The overview of applications of microfluidic POC devices in early diagnosis of cardiovascular diseases.

Classification	Detection Target	Composition	Sample	Comment	Ref.
Acute myocardial infarction (AMI)	Glycogen phosphorylase isoenzyme BB (GPBB), cTnT, and CK-MB	Paper-based microfluidic device	Serum	The platform has potential for the early diagnosis of AMI.	[43]
	cTnI, H-FABP, and copeptin	3D microfluidic paper analysis device (μPAD)	Serum	The developed platform has great potential for the early diagnosis of AMI.	[146]
Detection of multiple biomarkers for cardiovascular disease	C-reactive protein (CRP), troponin I (cTnI), and procalcitonin (PCT)	Paper-based microfluidic	Serum	The proposed immunosensor can be a great alternative for the early detection of cardiovascular diseases at the point of care.	[147]
	C-reactive protein (CRP), N-terminal pro b-type natriuretic peptide (NT-proB NP), cardiac troponin I (cTnl), and fibrinogen	Integrated microfluidic POC system with a field-effect transistor (FET) sensor	Serum	The sensor is promising for next-generation point-of-care devices assaying multiple CVDs biomarkers in clinical samples.	[148]

**Table 5:** The overview of applications of microfluidic POC devices in the early diagnosis of tumors (cancer).

Classification	Detection Target	Composition	Sample	Comment	Ref.
Cancer biomarker detection	Carcinoembryonic antigens (CEA) and alpha fetoproteins (AFP)	Fully Integrated hand-powered centrifuge and analysis paper-based microfluidic device (FICA μPADs)	Serum	The system successfully performed ELISA analysis of carcinoembryonic antigen and alpha fetoprotein from human blood samples.	[82]
	DNA methylation of tumor suppressor genes	PDMS microfluidic device	Cells, ascites, and serums	This developed microsystem may be promising for rapid and early diagnosis of cancers.	[157]
	CA-125 biomarker	A POC system combining a biochip and microfluidics	Blood through finger puncture	The system facilitates monitoring cancer progression and enables enhanced cancer management.	[160]
Lung cancer (LC)	Lung cancer specific exosomes	An integrative microfluidic device	Urine	The device has high sensitivity and specificity in isolating and detecting cancer-specific exosomes from patients’ urine.	[162]
Prostate cancer (PCa)	PSA concentration	System integrating dielectrophoresis (DEP), graphene field-effect transistors (FETs) and a compact disc–based microfluidic	Serum	The system was validated satisfactorily with commercially available existing systems using human serum samples.	[165]
Cervical cancer	HPV 16 and HPV 18	Paper-based microfluidic chip	Serum	This low-cost POC device requires less than 40 min to complete the test and has a low limit of detection.	[168]
Breast cancer	ERBB2	A microfluidic chip-based exosomal mRNA sensor	Serum	The system was proven to be effective for cancer diagnosis and liquid biopsies.	[169]

**Table 6:** The overview of applications of microfluidic POC devices in the early diagnosis of chronic diseases.

Classification	Detection Target	Composition	Sample	Comment	Ref.
Anemia	Hemoglobin concentration	Microfluidic system that uses optical methods	Serum	The system provides an approach that uses microfluidic detection of hemoglobin levels that can be integrated with other microfluidic approaches for blood analysis.	[179]
	Hemoglobin concentration	System integrating a 3D-printed microfluidic chip with a smartphone	Serum	This work presents a novel diagnostic strategy for advancing personalized medicine and mobile healthcare.	[180]
Chronic heart disease and chronic kidney disease	C-reactive protein (CRP)	System integrating a paper-based microfluidic immunoassay and a smartphone	Serum	The system has potential for future clinical POC chronic disease diagnosis and risk stratification through parallel measurements of a panel of protein biomarkers.	[181]
Advanced chronic kidney disease (CKD)	Phosphate concentration	Paper-based microfluidic device and a 3D-printed smartphone attachment	Serum	The device can potentially be used on a daily basis by patients at home.	[182]
Diabetes	Glycated hemoglobin	PDMS microfluidic device	Serum	The system enables earlier diabetes screening and diagnosis at a lower cost and earlier phase, minimizing the risk of diabetic complications.	[185]
		Paper-based microfluidic device using colorimetry	Saliva	Paper-based microfluidic devices have great potential for salivary diagnostics.	[189]
	Glucose	System integrating a paper-based microfluidic device and a smartphone	Serum	The paper-based microfluidic device is not susceptible to changes of sample volume.	[186]
		Paper-based microfluidic device using colorimetry	Saliva	Paper-based microfluidic devices have great potential for salivary diagnostics	[189]
Diabetes and hyperlipidemia	Glucose (GLU), triglycerides (TG) and total cholesterol (TC)	Smartphone-assisted microfluidic chemical analyzer	Serum	This study demonstrated the feasibility of performing multi-index monitoring of diabetes.	[190]
Early dry eye disease	The electrolytes in tears	Paper-based microfluidic device and a smartphone	Tears	The system demonstrates the feasibility for the detection of early-stage dry eye, differential diagnosis of dry eye sub-types, and the severity of the condition.	[62]

## Data Availability

Data will be made available based on request.
